# Insights into non-noble metal based nanophotonics: exploration of Cr-coated ZnO nanorods for optoelectronic applications

**DOI:** 10.1039/c7ra13174g

**Published:** 2018-02-12

**Authors:** Tejendra Dixit, I. A. Palani, Vipul Singh

**Affiliations:** Molecular and Nanoelectronics Research Group (MNRG), Discipline of Electrical Engineering, IIT Indore Indore Madhya Pradesh India vipul@iiti.ac.in; Mechatronics and Instrumentation Lab, Discipline of Mechanical Engineering, IIT Indore Indore Madhya Pradesh India

## Abstract

Herein, the room temperature photoluminescence and Raman spectra of hydrothermally grown ZnO nanorods coated with Cr are investigated for optoelectronic applications. A thorough examination of the photoluminescence spectra of Cr coated ZnO nanorods showed the suppression of deep level emissions by more than twenty five times with Cr coating compared to that of pristine ZnO nanorods. Moreover, the underlying mechanism was proposed and can be attributed to the formation of Schottky contacts between Cr and ZnO resulting in defect passivation, weak exciton–plasmon coupling, enhanced electric field effect and formation of hot carriers due to interband transitions. Interestingly, with the increase in sputtering time, the ratio of the intensities corresponding to the band gap emission and deep level emission was observed to increase from 6.2 to 42.7, suggesting its application for UV only emission. Further, a planar photodetector was fabricated (Ag–ZnO–Ag planar configuration) and it was observed that the dark current value got reduced by more than ten times with Cr coating, thereby opening up its potential for transistor applications. Finally, Cr coated ZnO nanorods were employed for green light sensing. Our results demonstrated that ZnO nanorods decorated with Cr shed light on developing stable and high-efficiency non-noble metal based nanoplasmonic devices such as photodetectors, phototransistors and solar cells.

## Abstract 2

Herein, the room temperature photoluminescence and Raman spectra of hydrothermally grown ZnO nanorods coated with Cr are investigated for optoelectronic applications.

## Graphical abstract

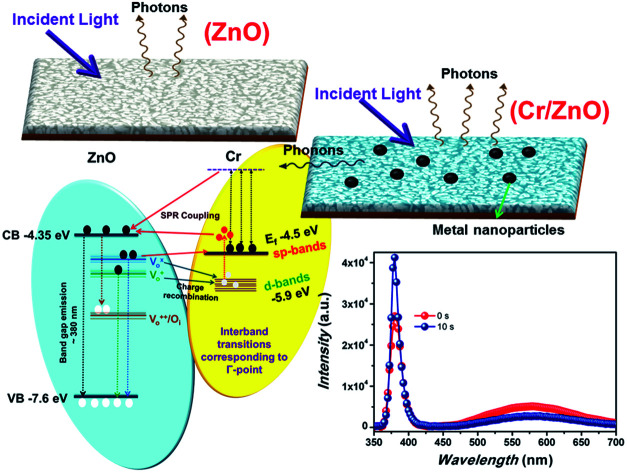

## 1. Introduction

In recent years, major attention has been given to the hydrothermal synthesis of ZnO nanostructures due to their variety, simplicity and tunability of the optoelectronic properties in comparison with other common oxide semiconductors *viz.* CuO, NiO, TiO_2_, FeO, SnO_2_*etc.*^1–6^ However, the biggest disadvantage of the hydrothermal growth of ZnO is the presence of defects which prohibits its potential application towards UV light emission and detection.^7^ It is well known that ZnO based nanostructures are strong candidates for UV emission and UV detection due to their wide band gap (∼3.37 eV) and large exciton binding energy (60 meV) at room temperature.^8^ Unfortunately, hydrothermally synthesized ZnO nanostructures are prone to feeble UV emission and dominant deep level emissions (DLE), therefore passivation of DLE and subsequent UV emission enhancement remains a great challenge towards the fabrication of next generation optoelectronic devices.^7^ The enhancement in the near band edge emission (NBE) in terms of the UV-to-Vis emission intensity ratio has therefore become one of the vital issues in the research of ZnO for its potential applications in the field of short wavelength semiconductor lasers and light emitting diodes.^9,10^ In order to selectively enhance the UV emission and simultaneously reduce DLE towards the UV only emission, numerous techniques have been applied in recent years by scientific community. Among them, the most effective methods are (1) addition of oxidizing agents like KMnO_4_, K_2_Cr_2_O_7_, H_2_O_2_ in the precursor solution during the growth, (2) dielectric coating *viz.* PMMA, polyaniline, Al_2_O_3_, ZnS, MgO, (3) plasma treatment, (4) thermal annealing in the Ar environment, (5) post growth treatment with H_2_O_2_ and most importantly (6) metal coating on ZnO thin films and nanostructures.^11–21^ Much effort has been focused in the area of improved UV emission of ZnO thin films and nanostructures using metal capping since Okamoto *et al.* demonstrated in their leading work the dramatic emission enhancement from the Ag-capped InGaN/GaN quantum well.^22^ The primary reason behind the emission enhancement is surface plasmon (SP) resonance arising due to the local electric field enhancement near the surface of the metal nanoparticles.^22,23^ There are several reports on enhanced PL emission of ZnO nanostructures with the use of noble metals like Au, Pt and Ag.^24–26^ However, the exorbitant price of these noble metals restricts their utility for SPR enhanced sensors/optoelectronic devices. Thus there is a need to use some practical metals instead of these noble metals for localized surface plasmon resonance (LSPR) effect. A possible approach to circumvent the problem is coating ZnO nanostructures with transition metals with the purpose to enhance their radiative efficiency by utilizing the interaction of excitons in ZnO and SP arising from collective electron oscillations in the transition metals.^27^ Such SP mediated emission has recently been demonstrated in ZnO nanostructures coated with various metals (Al, Ni, Ti, Zn and Cu) and substantial enhancement of the NBE emission was observed.^28–32^ Hence, extending plasmonic properties to non-noble metals, most specifically, transition metals would definitely open up the range of applications in the field of optoelectronics. Interestingly, it should also be possible to realize SP–exciton coupling in Cr capped ZnO nanostructures, as transition metals support SP modes at their surfaces.^27,33^ Despite the fact that the proof of this concept may lead to promising applications of ZnO/Cr systems in optoelectronic devices, no work on this issue has so far been reported in the literature. Being an economic metal its effect towards tuning the optoelectronic properties of ZnO NRs calls for rigorous analysis. The purpose of this work is to understand the effect of Cr coating and to explore the SP–exciton coupling by means of optical spectroscopic techniques.

Another aspect of utilization of ZnO nanorods (NRs) towards next generation optoelectronics covers enhanced emission and detection of visible light.^34,35^ Furthermore, the outstanding visible luminescent performances of ZnO nanostructures, can therefore be utilized for visible light emitting devices, optoelectronic devices, energy storage and conversion.^36–38^ Apart from UV emission, ZnO also exhibits violet, blue, green, yellow, orange and red emission *i.e.*, all the major constituents of white light.^7^ Although, the hydrothermal technique is inherent to defect related emissions, however a systematic way to control the DLE is highly desired. In order to improve the visible emission in a controlled way many techniques like (1) coating with graphene oxide sheets, (2) ZnO growth in Zn-rich conditions, (3) pre-induced oxygen deficient environment *etc.* have already well utilized by scientific community.^39,40^ Here in this article, we have shown a systematic enhancement in the visible emission peak (simultaneous suppression of UV emission) with the thermal annealing treatment of Cr coated (with increasing sputtering time) ZnO NRs, thereby extending its coverage in the visible spectra also. Diffusion of Cr in the ZnO lattice and corresponding occupation of oxygen vacancies can be assigned for improved visible light emission. Additionally, improved green light sensing was also observed with Cr coating which is difficult to observe in case of pristine ZnO NRs. Most importantly the reduction in the dark current level by more than 10 times has been observed for Cr coated ZnO NRs, which thereby suggest its application for photosensitive transistors as they require low dark current in order to improve the ON/OFF ratio. The work presented here carries tremendous potential for ZnO based luminescent materials and photodetectors towards the fabrication of next generation optoelectronic devices.

## 2. Experimental procedure

The ZnO NRs were fabricated on glass substrate *via* simple hydrothermal technique as described in our previous reports.^11^ In brief a seed layer solution (2 M solution of zinc acetate and ethanolamine in 2-methoxyethanol) was spin coated (3000 rpm, 30 s) for seed layer growth of ZnO and were annealed at 250 °C in order to improve the adhesion of seed layer with the substrate.^11,12^ Subsequently, a solution of equimolar (0.1 M) zinc nitrate hexahydrate and HMTA in DI water was prepared. In the next step, 2.5 mM KMnO_4_ was added and the solution was stirred before sample immersion and then kept at 110 °C for 4 h for nanorod growth.^11^ The deposition of Cr ((99.99% purity) purchased from ACI Alloys, USA) on these ZnO NRs was finally carried out by using rf-magnetron sputtering system (60 W RF power at 10 sccm Ar flow). The sputtering time of Cr coating was varied from 0–200 seconds in order to get the complete overview of the effect of Cr coating.

The morphology and structure of the products was observed by a field emission scanning electron microscope (FESEM), Zeiss Supra-55 equipped with energy dispersive spectra (EDS) (Oxford Instruments, X-MAX, 51-XMX1025). Powder XRD (Rigaku Smart Lab® system) with CuKα radiation operating at 40 kV and 40 mA, with wavelength (*λ*) = 1.5418 Å was used to examine the crystallinity and phase information. The PL spectrometer (Dongwoo Optron DM 500i) having an excitation source consisting of a continuous wave He–Cd laser (excitation wavelength, 325 nm, PMT detector) was used to measure the PL emission from these samples at 295 K. Confocal microscope images were obtained by using a Leica TCS SP5 confocal microscope equipped with PMT detector at room temperature. Moreover, the images were obtained by using an Olympus BX51 TR-N33MU optical microscope. In our experiment, Raman spectra (RIR-M151) were recorded at room temperature under the 530 nm excitation (laser power of 7.5 mW).

For fabricating the photodetector, 100 nm thick lateral electrodes of Ag were then deposited onto the ZnO NRs by thermal evaporation (HHV-PVD system), which served as ohmic contacts. The spacing between Ag electrodes was nearly kept to be 500 μm by employing shadow mask technique. The active area of the entire device was 0.5 × 0.5 mm^2^. Electrical and photodetection characterization of the planar photodetectors was performed using a Keithley 2612A dual channel source meter unit. All measurements were performed at room temperature.

## 3. Results and discussion

### 3.1 Morphology and structural analysis

**Fig. 1:**
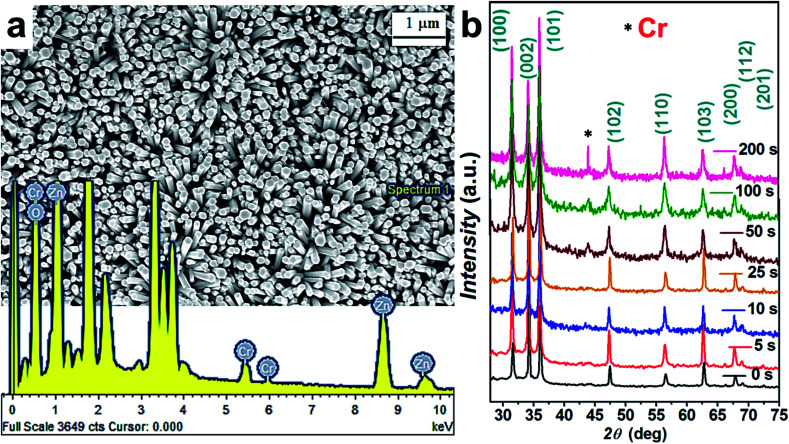
(a) FESEM images for ZnO nanorods grown with the addition of 2.5 mM KMnO_4_ in the precursor solution, (b) XRD plots for various samples, the inset shows the EDX spectra of Cr coated ZnO nanorods.

Fig. 1(a) show the typical top-view FESEM images of the hydrothermally grown ZnO NRs with the addition of 2.5 mM KMnO_4_ in the precursor solution. Interestingly, vertically oriented NRs (with average rod diameter of 64 nm) have been observed, the detailed mechanism of which has been already reported and well discussed in our previous works.^11^ Concisely, KMnO_4_ play significant role while controlling the growth rate, orientation and defect states by providing OH^−^ ions and O_2_ in the precursor solution.^11,12,41^

XRD plots for Cr coated ZnO NRs are shown in Fig. 1(b). A dominating (002) peak along with (100), (101), (110) and (112) was seen for all samples.^11^ The ZnO NRs and the Cr coated ZnO NRs exhibit the same XRD spectra, the only difference being the diffraction intensity which depends on the metal undercoating. The presence of Cr nanoparticles is found to induce a reduction in the diffraction intensity corresponding to (002) peak due to the presence of Cr nanoparticles role of impurities induced defects in the ZnO matrix.^42^ As shown in Fig. 1(b), for all of the deposited samples, the Cr (110) diffraction peak was present in the XRD patterns. The proper position of observed Cr diffraction peak is in agreement with Joint Committee for Powder Diffraction Standards (JCPDS) standard data (refer to JCPDS card no. 088-2323). No characteristic peaks of other phases were observed in the samples. An EDX spectrum of the samples is shown in the inset of Fig. 1. Presence of Cr can be clearly seen from the EDX spectra.

### 3.2 Optical characterization

**Fig. 2:**
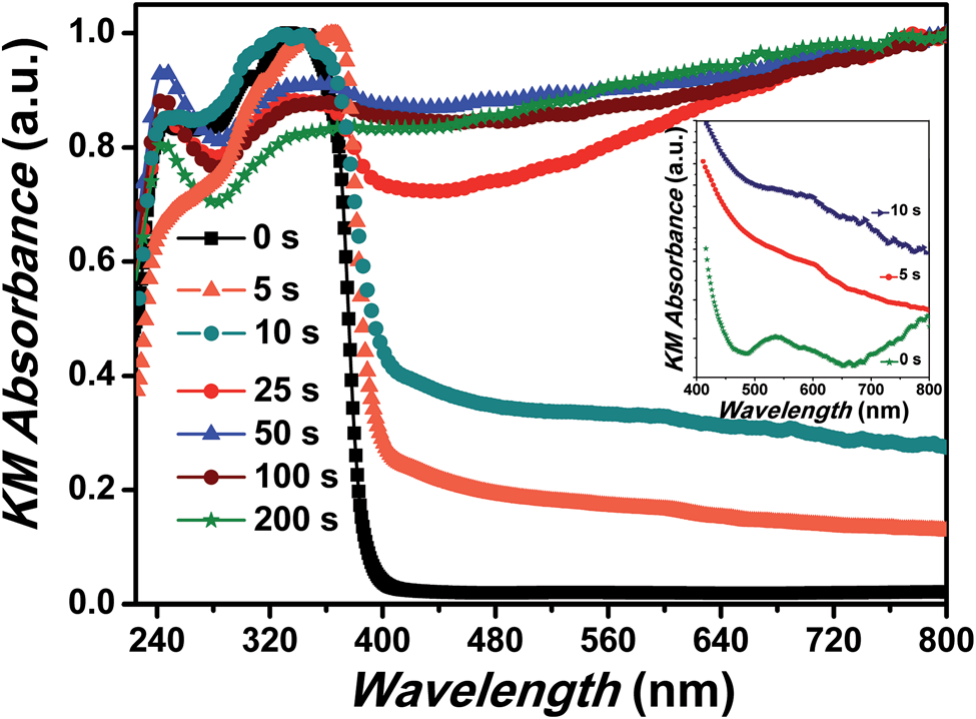
Absorption spectra of pristine and Cr coated ZnO nanorods for various sputtering time durations from 0 s to 200 s. The inset shows the optical absorption spectra of pristine and Cr coated ZnO nanorods in the visible region due to defects.

In order to systematically analyse the effect of Cr coating on the ZnO NRs, Kubelka–Munk (KM) absorption spectra was recorded and has been shown in Fig. 2. In general the absorption spectra of ZnO nanostructures comprise one strong band in the UV region and diminished absorption in the visible region of the electromagnetic spectrum.^43^ Interestingly, here for pristine ZnO nanorods two separate bands (∼335 nm and 346 nm) in the UV region were clearly observed. In ZnO, the crystalline field and the spin–orbit interaction lift the valence band degeneracy at the *Γ*-point of the Brillouin zone.^44^ More precisely, from the three valence sub-bands and the conduction band, one can build three exciton series labelled A, B and C.^45^ The high energy peak can be assigned to the C-exciton and the low energy line to the sum of the contributions from the A and B excitons.^41^ Such a clear splitting in the excitonic bands normally appears in the ZnO nanostructures with low density of defects.^44–46^ In nanostructures with low-crystallinity and high density of defects, these peaks are broad and usually overlap with each other forming a single broad absorption band in the UV region. Additionally, a broad and feeble absorption band ranging from 485 to 650 nm in the visible region with peaks at 534 and 590 nm have been observed, which is actually responsible for green, orange and red emissions in ZnO NRs (shown in the inset of the Fig. 2). The presence of charge on the oxygen vacancies (mainly 
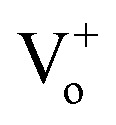
 and 
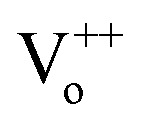
) and large dispersion of the hybridized sp-orbitals are the major reasons behind the weak optical absorption in the visible region.^47^ These results are actually different from those generally obtained in most ZnO nanostructures, in which no absorption band appeared in the visible region. These absorption peaks can be assigned to several physical processes in the NRs like singlet excitation in ionised oxygen vacancy, zinc interstitial or antisite oxygen.^48^ It has already been mentioned that KMnO_4_ addition increases the content of oxygen in the precursor solution and thus reduction in the defect density has been observed.^11^ Here for pristine ZnO samples not only do the curves show different slopes but some curves are also asymmetric, in a manner that the absorption band extends well into the low photon energy regime. Further, the Urbach energy for ZnO samples was analysed and the value was calculated to be 40.3 meV. Very low Urbach energy for pristine ZnO samples clearly demonstrates better optical quality of ZnO NRs in comparison to the usually grown ZnO nanostructures, which has already been observed in XRD results.^49^

Interestingly, with Cr coating the absorption edges has been observed to be red shifted by more than 25 nm (from 350 nm to 376 nm after Cr coating). The red shift and broadening has indicated energy transfer between Cr and ZnO NRs.^41^ It is important to note here that Cr typically shows optical absorption between 1–2 eV (with a shoulder at 2.31 eV) which can be assigned to the interband transitions (1.56 eV) around the *Γ* point while the shoulder absorption band is due to the transitions from the *Σ*-axis respectively.^50–52^ Along with these two bands another transition from *D*-axis as well as from *Λ*-axis also contributes to the absorption spectra with peak at 3.2 eV.^50^ The optical absorption of Cr constitutes Drude (intraband transition due to electron–phonon collisions) and interband absorptions.^27^ A reasonably strong and broad interband transition in Cr is localized in a wide energy range around ∼1.56 eV and can be attributed to the direct transitions originating from 
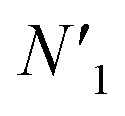
 and 
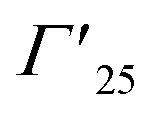
 symmetry points.^51^ The pronounced interband transitions can therefore be assigned to the transfer of oscillator strength from the intraband to the interband modes. Moreover for visible and NIR radiation interband transitions are much larger than the intraband transitions hence the dominating mechanism is interband transition only owing to the multi-electron effect.^41,53,54^ Interestingly, for transition metals with partially filled d-bands (hence for Cr also), intraband transitions will be strongly damped by the interband transitions, which play a major role in the determination of the optical response.^27^ The broad absorption band ranging from 550 to 800 nm can be assigned to the interaction between ZnO defect states and interband transitions of Cr as can be seen from Fig. 2. Therefore the interaction between ZnO defect states and these two bands can be a possible reason for such a broadening in the visible region absorption. Generally, the width of optical absorption band related to interband transitions (depends on the width of d-band) is expected to play an important and integral role while determining the optical response. Additionally, lattice disorder as well as surface scattering can also contribute for broaden absorption band. The absorption band in the range of 550–800 clearly supports the interband transitions around 1.5–2 eV which thereby produces hot holes and hot electrons.^55^ These hot carriers can play as significant role in the tuning of the optoelectronic properties of ZnO NRs.

**Fig. 3:**
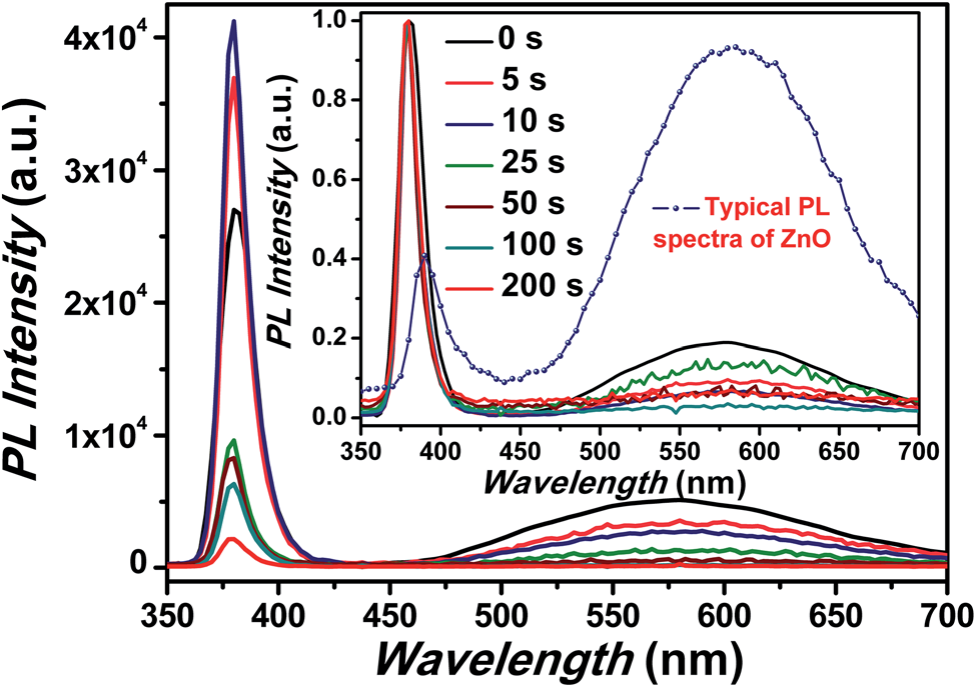
PL spectra of pristine and Cr coated ZnO NRs. The inset shows the normalized PL spectra. Inset also shows the typical PL spectra of ZnO grown using hydrothermal process.

The room temperature PL spectra corresponding to ZnO NRs coated with Cr with different sputtering time using rf-sputtering has been illustrated in Fig. 3. For bare ZnO NRs, a band gap emission around 380 nm with a full width at half maxima (FWHM) of 18.6 nm (due to exciton recombination) and a broad emission band around 577 nm were observed in the PL spectra. It has been recognized that in ZnO NRs the broad PL band at 577 nm is related to transition of electrons from oxygen vacancies or shallow donors to Zn vacancies, or other intrinsic defects such as oxygen interstitials.^11^ This weak and broad PL band has been assigned to deep level emissions (DLE). It must be noted here that the ratio of the intensity of the peaks corresponding to NBE to that of DLE (*I*_NBE_/*I*_DLE_) provides a glimpse of the optical quality of the samples.^56^ Here the value of *I*_NBE_/*I*_DLE_ was observed to be five for pristine samples indicating intense NBE, which is higher than the typical value (less than one) of *I*_NBE_/*I*_DLE_ for the samples grown without any addition of surface modifiers and/or additives, as the hydrothermal process is prone to feeble UV emission (shown in the inset of Fig. 3). Interestingly, after Cr coating an increase in the value of NBE emission intensity and simultaneous suppression of DLE has been observed. Here it must be noted that the work function of Cr is −4.5 eV *i.e.* below the ZnO conduction band.^57,58^ Therefore theoretically, it should make Schottky contact with ZnO (conduction band level −4.3) and thus both NBE and DLE should suppress due to the reduced radiative recombination as the band bending will prohibit the flow of electrons thereby suppressing the emission, however contrary to this we have observed an increase in NBE and subsequent suppression in DLE. Not only, can it be related to coupling between the spontaneous emission of the NBE excitons in ZnO and SPs of the Cr metal but also to the enhanced electric field effect. As surface electromagnetic waves can exist due to Cr nanoparticles, hence the involvement of plasmonic effects and plasmon–exciton coupling cannot be ignored.^27,59^ The utilization of plasmonic effects of other non-noble metals like Ni and Ti has been already reported in the literature and has been shown to have great influence on the emission characteristics of metal coated ZnO nanostructures.^29,60^ While Cr is having similar optical properties as that of Ni in the UV and visible region along with the plasmon–exciton coupling therefore, it can be provide a reasonable solution to the aforementioned problem.^61^ This SP–exciton interaction at the metal/ZnO interface may occur when the SP energy is comparable to the ZnO band gap energy. Interestingly, for Cr the surface plasmon resonance usually occurs in the UV region (∼300 nm), hence it can couple with excitons got generated in ZnO (∼380 nm) under UV illumination.^27^ Noteworthy is the fact that the FWHM of the NBE peak was observed to be strongly dependent on the Cr sputtering time. Precisely, the value decreases from 18.6 nm to 12.8 nm for pristine to Cr coated ZnO nanorods for 25 s deposition using sputtering suggesting reduction in non-radiative recombination rate.^48,62^ Additionally, an increase in the FWHM values was observed after increasing the sputtering time beyond 25 s (as shown in Table 1) simply signifying formation of trap states and a significant increase in the non-radiative recombination rate.^48^

**Table 1:** Comparison of UV and visible emission peak positions (nm) for Cr coated ZnO samples with varying sputtering time. Full width at half maximum (FWHM) for NBE peak is shown in fourth column. All the values are shown in ‘nm’ scale

Sputtering time	UV peak position (nm)	Visible peak position (nm)	FWHM of UV peak (nm)
0 s	380.1	576.9	18.6
5 s	380.0	586.1	13.0
10 s	379.8	589.9	13.8
25 s	379.8	590.1	12.8
50 s	378.9	590.0	13.4
100 s	378.8	594.2	13.5
200 s	378.6	592.4	14.4

It is interesting to note here that, with Cr coating, suppression in DLE with peak position mainly in the orange region of the visible spectra has been observed. The DLE peak positions are mentioned in the Table 1 and have indicated strong red shift from the peak position corresponding to the pristine sample. This gradual red shift of the DLE peak position with Cr sputtering time clearly indicates that 
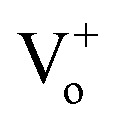
 and 
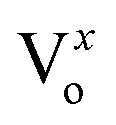
 states got passivated significantly, while Cr coating does not play significant role for the passivation of defect states belonging to the doubly ionized oxygen emissions (
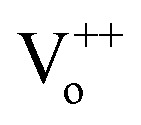
).^63^ In general, the suppression of the DLE has been attributed to the transfer of electrons from defect states to the metal Fermi level and coupling between excitons and surface plasmons.^19^ Although this mechanism seems satisfactory for metals having work function below to the defect states of ZnO.^41^ Now, it is worthwhile to note here that Cr forms Schottky contacts with ZnO, hence the ZnO conduction band level (4.3 eV) will lie above to the Cr Fermi level (4.5 eV), thereby theoretically resulting into the band bending at interface which would definitely suppress the NBE and DLE emission.^23^ However, the contradictory observation of the enhancement of NBE in the PL spectra supports our assumption of plasmon–exciton coupling. It is important to mention here that the existence of plasmon resonance in Cr is highly related to the interband transitions and therefore get affected by it. As can be seen from Fig. 3 that the NBE emission enhancement ratio is nearly 1.5 for Cr coated (10 s) ZnO NRs. Although there is an enhancement in the value of the NBE however it is lower than the typical values of the enhancement ratio that were reported for other metals like Au, Ag, Pt and Al.^25,26,64,65^ It is worth to mention here that the exciton–plasmon coupling strength depends prominently on the magnitude of the real part of dielectric constant of the metal (the real part of the dielectric constant of the metal must be negative and its magnitude must be greater than that of the semiconductor). For Cr, the magnitude value of the real part of dielectric constant is not as large as for the other plasmonic metals like Ag, Au and Al in the UV and visible region of the electromagnetic spectra, therefore, it is expected that the effect of plasmon coupling will be less pronounced in the case of Cr.^61^ However, the plasmon–exciton coupling efficiency can be remarkably modified by varying the shape and size of nanoparticles of metals. Hence, the plasmon resonance peak and thus the coupling between plasmons and excitons can be improved significantly with the variation of size and shape of Cr NPs.^27^

In order to provide a plausible justification of the above mentioned results, we have proposed the involvement of hot carriers either generated through the decay of surface plasmons or interband transitions as a dominating contributor for the NBE enhancement and DLE passivation.^55,66^ Illumination of metallic nanoparticles with appropriate sources produces strong optical near-fields that initiate a series of processes with multiple outcomes, including the excitation of surface plasmons, their radiative decay to photons and non-radiative decay into hot carriers.^67^ These carriers are considered as hot carriers, since these have significantly large energy than those of thermal excitations at ambient temperatures.^68^ Further, these carriers can be injected into semiconductor as Landau damping of the collective oscillation of these electrons can result into highly energetic carriers and therefore can significantly increase the probability of transfer of these hot carriers into the semiconductor.^69^ Hot carrier generation through non-radiative decay of surface plasmons at metal semiconductor interface can occur either through direct or phonon assisted transitions. This process is typically allowed above the interband threshold energy of the given metal.^70^ Interestingly, in the case of Cr the direct transition occur from the d-band to the unoccupied state above the Fermi level, which results in generation of hot holes (in d-bands), which usually are ∼1–2 eV more energetic than the hot electrons.^27^ Subsequently, upon generation plasmons undergo fast de-phasing with the creation of electron–hole pairs. Plasmon de-phasing comes from collisions with phonons and defects along with electron–electron scattering. There is a significant probability of non-radiative decay of these plasmons, thus the fluorescence quantum yield is very low.

**Fig. 4:**
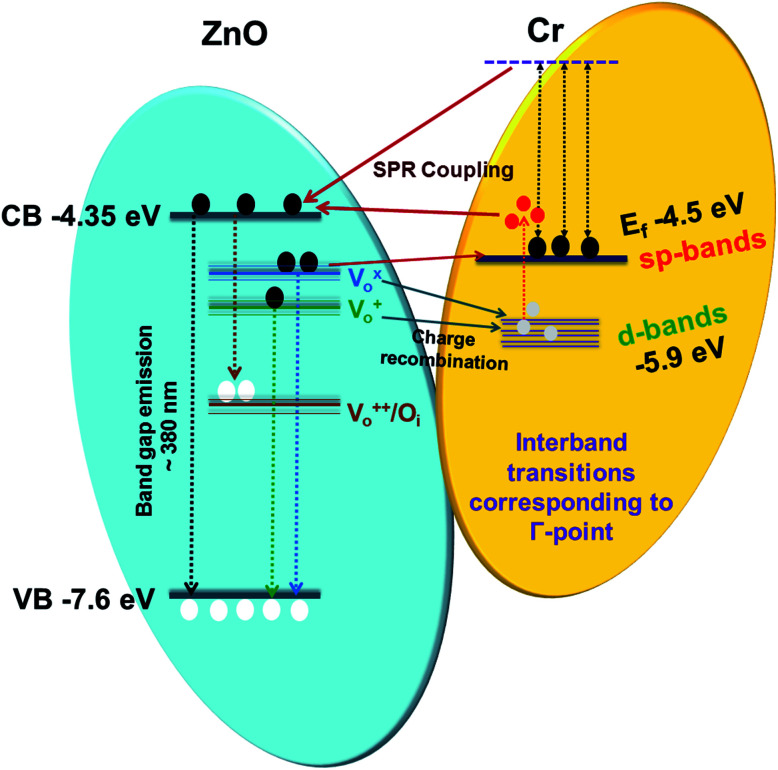
Schematic diagram for the mechanism of the emission enhancement in Cr coated ZnO NRs.

It is interesting to mention here that plasmon resonance also takes place in Cr as have been mentioned earlier in the absorption spectra of Cr film (transitions from the Fermi surface to the flat unoccupied band). Thus, both surface plasmon decay and interband transitions will generate hot holes in the d-bands and hot electrons in sp bands. This hot interband electron–hole pair generation can lead to the hot electron injection into the CB of ZnO NRs thus will enhance the NBE emission. Interestingly the hot holes can easily recombine with the electrons present at the 
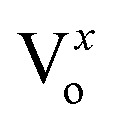
, 
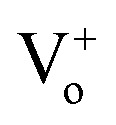
 states, which results into the suppression of DLE corresponding to the blue and green region of visible spectra. It can be clearly seen that the hot hole levels are actually below to the ZnO defect levels, and thus can provide energetically favourable path for recombination of the electrons present at the defect levels with hot holes further leading to the suppression of defect related emissions primarily in the blue and green region of the emission spectra of ZnO. In addition to this, hot electrons can directly go to the ZnO conduction band level which could lead to NBE enhancement along with the contribution due to plasmonic transitions and exciton–plasmon coupling. Thus it can be stated that hot electrons can play major role in NBE enhancement while hot holes in DLE passivation in the case of Cr coated ZnO NRs. Strikingly, hot carrier generation through interband transitions play a major role in comparison to that of the hot carrier generation through plasmon decay.^27^ The schematic of the above mentioned mechanism is shown in Fig. 4.

**Fig. 5:**
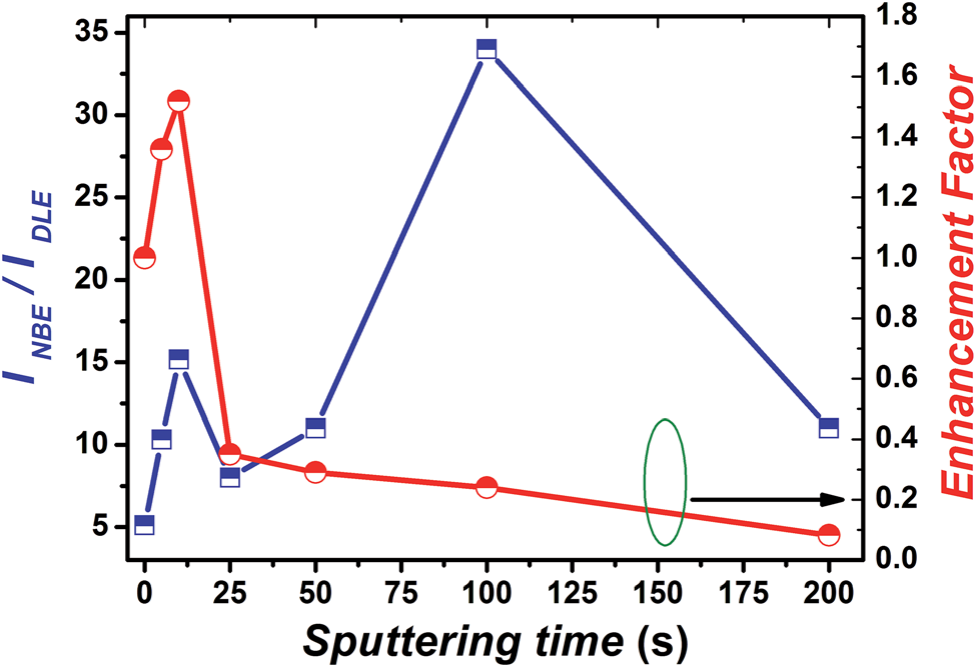
Dependence of the ratio of PL intensity corresponding to NBE and DLE and enhancement factor on the Cr sputtering time.

**Fig. 6:**
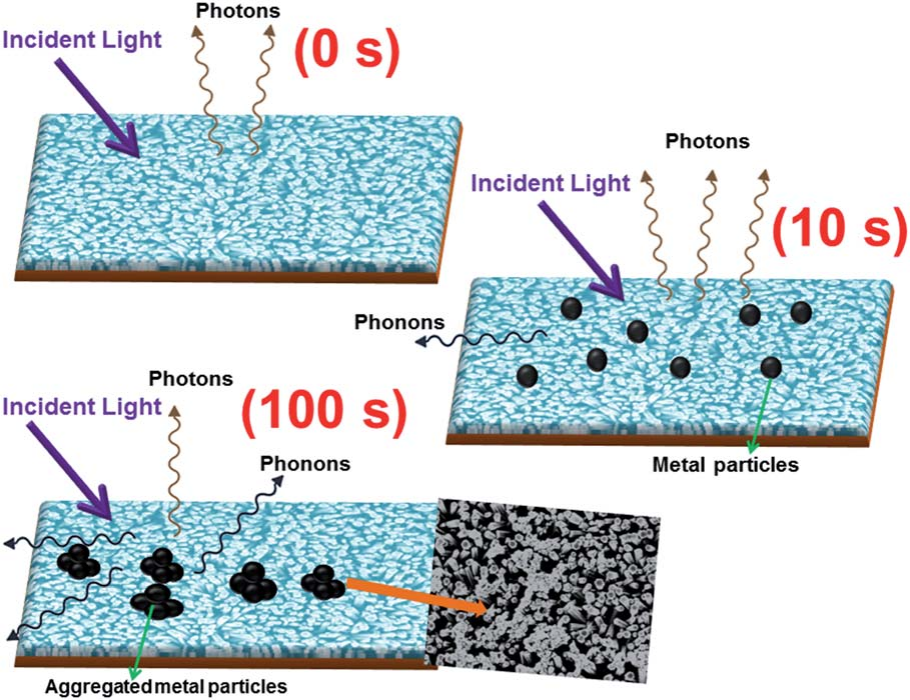
Schematic diagram for the mechanism of the involvement of phononic transitions on the emissions of pristine and metal coated ZnO NRs (10 and 100 s).

To gain better understanding of the mechanism of the above mentioned phenomenon, we have calculated the enhancement ratio for Cr-coated ZnO NRs with different sputtering time. From Fig. 5, it has been observed that the enhancement ratio start increasing with the increase in the Cr-sputtering time. Moreover, it reached to a maximum value of nearly 1.5-fold for a sputtering time of 10 s. Surprisingly, it starts to decrease subsequently for sputtering times over 10 s. The increase of enhancement ratio can be simply attributed to the increased coupling between the DLE of ZnO with the SPs of Cr NPs and hot carriers at the interface. It is obvious to interpret that the plasmon–exciton coupling strength increases with increasing coverage of Cr NPs on ZnO NRs. Plasmon–exciton coupling is highly sensitive to metal shape, size and density and can be controlled by the sputtering time. Capping Ag films on InGaN/GaN quantum well and Au-coated films on ZnCdO thin films for enhancement of spontaneous emission have been already demonstrated by scientific groups.^71,72^ It was clearly shown by Zhang *et al.* that there exist an optimum density of metal NPs for best value of enhancement of PL^71,72^ Therefore in the case of Cr also there should be a minimal density of Cr NPs for obtaining the maximum enhancement ratio as can be seen from Fig. 6. Generally, the trade-off between absorption, scattering and plasmon coupling of metal NPs play a critical role in the governance of plasmon–exciton coupling. When the density of Cr NPs becomes larger than the trade-off density of the metal NPs, absorption of Cr NPs and phononic transitions at the interface could gradually dominate the process.

In general, photo-generated excitons in semiconductors decay through both radiative and non-radiative recombination processes as have been shown in Fig. 6. Mathematically, the PL decay time (*τ*_ZnO_) of bare ZnO NRs can be therefore expressed as:
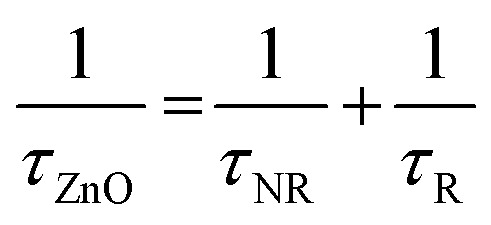
where, *τ*_NR_ and *τ*_R_ represent the non-radiative and radiative decay time of pristine ZnO NRs, respectively.^73^ Surface defects of ZnO play a major role in determining *τ*_R_ and *τ*_NR_. In our previous section we have shown the passivation of surface defect with KMnO_4_ addition in the precursor solution, therefore we have observed a feeble DLE suggesting higher radiative recombination rate. Interestingly, after decorating the ZnO NRs with Cr NPs, additional radiative recombination resulting from the coupling will also govern the PL decay of Cr coated ZnO NRs.
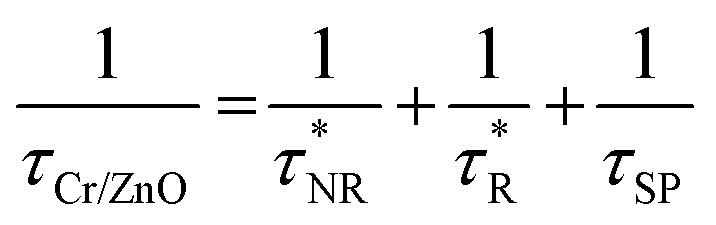


Thus, the PL decay time (*τ*_Cr/ZnO_) will decrease due to plasmon coupling to some extent and can be expressed as 
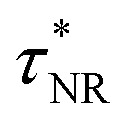
 and 
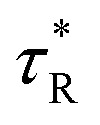
. Where, 
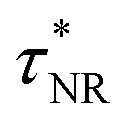
 and 
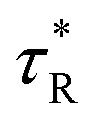
 are the non-radiative and radiative decay times of Cr coated ZnO NRs, respectively. 1/*τ*_SP_ is the plasmon–exciton coupling rate.^74^ A significant decrease in the PL decay time can be expected for Cr/ZnO primarily due to the defect-SP coupling, compared to that of the pristine ZnO NRs.^75^ The above mentioned qualitative analysis have suggested that the *τ*_Cr/ZnO_ will gradually decrease with increasing density of Cr particles over ZnO NRs as shown in Fig. 6. Alternatively, the stronger the local SP field became, the more would be the probability of the scattering by the rough surface of Cr. Thus, the coupled energy could become significant, thereby exciting the more coupled SP polaritons into free space to take part in radiative recombination *via* scattering (Fig. 6).^74^ With Cr sputtering time of more than 10 s, we deduced that some Cr NPs began to coalesce together. This is a typical Ostwald ripening, as shown in Fig. 6 for the case of Cr coating for 100 s. At this stage the intense absorption would dominate the scattering process, leading to an enhancement of non-radiative recombination. This increased non-radiative recombination rate also supports the previously observed low enhancement ratio in the case of Cr coating in comparison to other metals (as has been observed from the PL spectra). Additionally, capping of ZnO surfaces with metal films may cause a reduction of density of surface recombination centers consequently can lead to an increase of the NBE emission. Therefore, the probabilities of radiative trap recombination at the surface defects will decrease when the surface is passivated by capping a metal layer which will eventually, decrease the probabilities of radiative trap recombination.

**Fig. 7:**
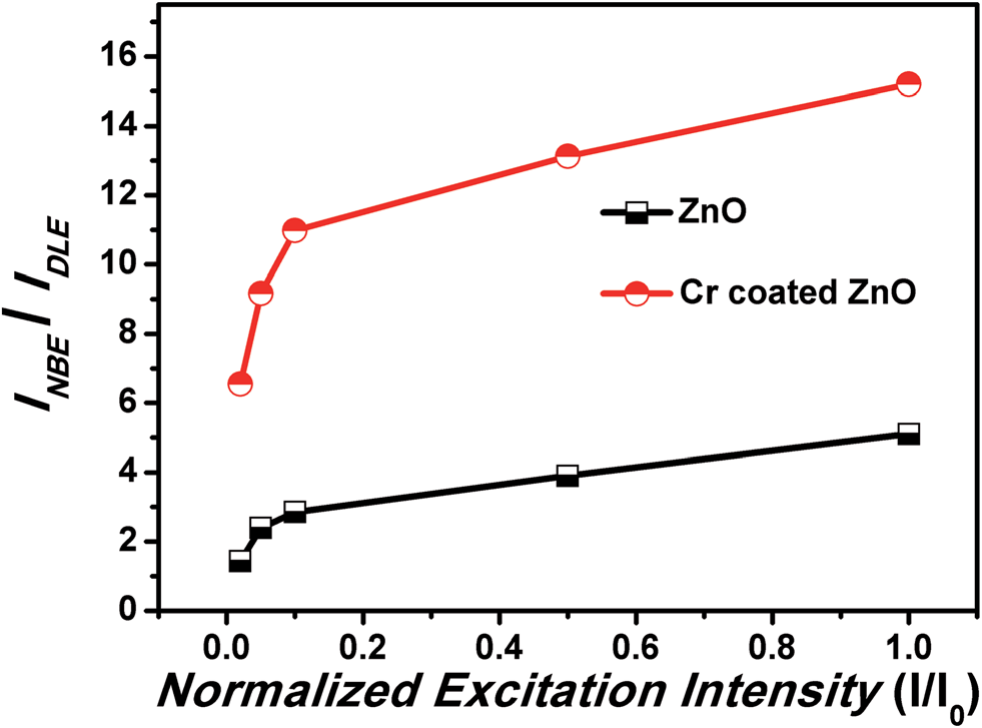
Dependence of the ratio of PL intensity corresponding to NBE and DLE and enhancement factor on the excitation intensity.

Excitation intensity dependent PL spectra can also shed some light on the effect of Cr coating over the passivation of defects. Dependence of the emission spectra of as-synthesized ZnO samples on the laser excitation intensity has been shown in Fig. 7. It has been established that the luminescence intensity *I* can be expressed as:*I* = *ηI*_0_^*α*^

In this relation *I*_0_ is the power of the excitation laser radiation (10 mW in this case), *η* is the emission efficiency and the exponent *α* represents the type of radiative recombination mechanism.^62^ For the excitation-intensity variations of the NBE emission intensity acquired at 293 K, the exponent *α* has a value at around 1.5 for the pristine samples and 1.68 for Cr coated ZnO NRs, hence indicating more enhanced band gap emission with Cr coating.^62^ The increase in the value of *α* is an indication of passivation of defects. Moreover, a blue shift of 4 nm, in the UV peak positions was observed, therefore the effect of laser induced heating can be discarded in both cases. In order to have a thorough insights into the effect of excitation intensity on the Cr coated ZnO NRs PL, we have analysed the variation of *I*_NBE_/*I*_DLE_*vs.* excitation intensity (shown in Fig. 7). A significant change has been observed in *I*_NBE_/*I*_DLE_ with the variation of excitation intensity. A significant decrease in the value of the *I*_NBE_/*I*_DLE_ has been observed at low excitation intensities for the case of as synthesized ZnO NRs as well as for the Cr coated ZnO NRs.

**Fig. 8:**
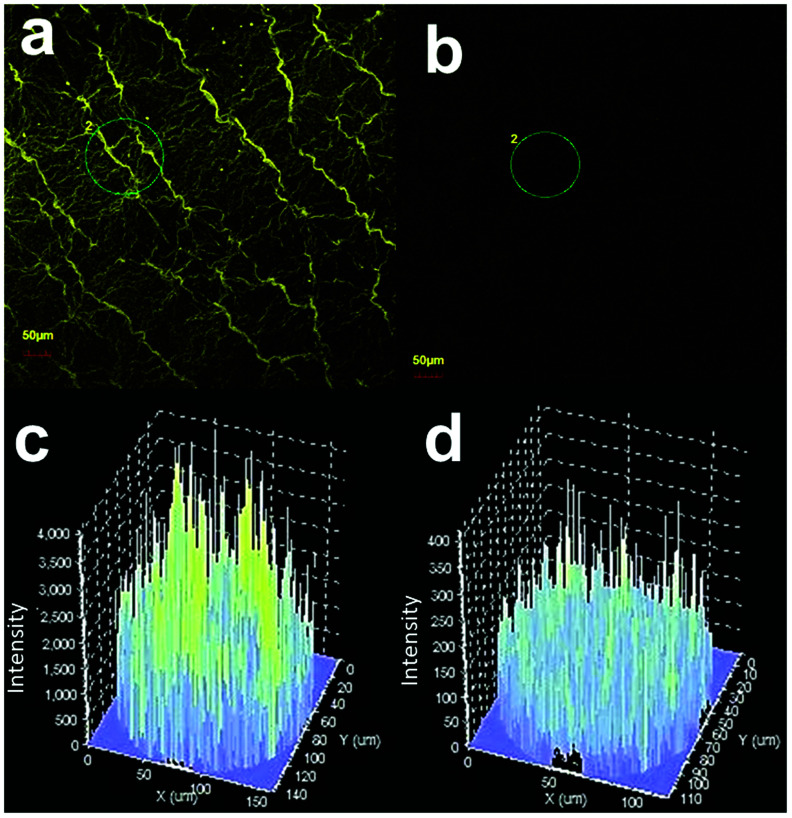
Confocal microscopy images of the pristine and Cr coated ZnO samples.

Fig. 8(a) shows confocal-microscope images of the pristine ZnO NRs (under illumination by a 488 nm laser) captured over the range of 500–650 nm in order to systematically analyze the DLE. The confocal-microscope measurements were conducted to investigate the variation in the fluorescence intensity mainly in the visible region due to the coating of Cr layer over the ZnO NRs.^35^ The confocal-microscope images (Fig. 8(b)) show a suppressed DLE from the ZnO NRs coated with Cr using sputtering. The intensity of the selected regions has been shown in Fig. 8(c and d) for pristine ZnO NRs, and Cr coated ZnO NRs. The intensity values for Cr coated ZnO NRs were nearly at noise level and were in well resemblance with PL results.

Wurtzite structured ZnO belongs to *P*63*mc* symmetry group and consists of two zinc and two oxygen atoms in a unit cell with twelve phonon modes (of which nine are optically active).^76^ At *Γ* point of reciprocal space, phonon modes are represented as:*Γ*_out_ = *A*_1_ + 2*B*_1_ + *E*_1_ + 2*E*_2_

**Fig. 9:**
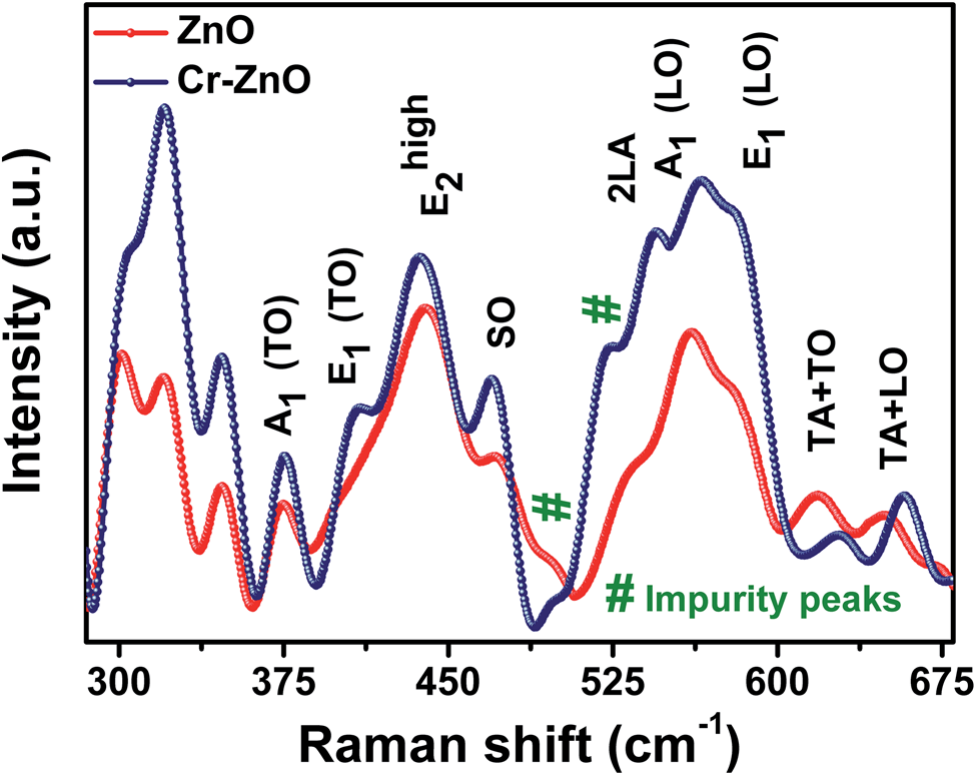
Raman spectra of pristine and Cr coated ZnO NRs. Here Cr was sputtered for 10 s using rf-sputtering.

In wurtzite ZnO crystals, the non-polar phonon modes with symmetry *E*_2_ have two frequencies, *E*_2_ (high) is associated with oxygen atoms while *E*_2_(low) is associated with Zn defects.^77^Fig. 9 shows the Raman spectra of pristine and Cr coated ZnO samples (for 10 s). The usual modes in ZnO, such as 333 cm^−1^ (*E*_2_(high) − *E*_2_(low)) and 437 cm^−1^ (*E*_2_(high)), were observed.^56^ The peak at 375 cm^−1^ can be assigned to A_1_(TO) mode.^78^ In addition to this a peak was observed at 410 cm^−1^ and can be attributed to E_1_(TO) modes. The peak at 500 cm^−1^ can be attributed to the disorder activated Raman scattering of silent mode 2-B_2L_.^56^ Moreover, the peak at 579 cm^−1^ has been observed and can be assigned to the longitudinal optical (LO) mode.^29^ The 579 cm^−1^ band *i.e.* E_1_(LO) mode can be assigned to defect states like, oxygen vacancies, Zn interstitials. It is well reported that this peak has strong dependence on the surface electric field and hence can be modulated using metal NPs.^79^ Interestingly both broadening and increase in the intensity of the peak corresponding to E_1_(LO) mode was observed in case of Cr coated ZnO NRs (Cr coating for 10 seconds), clearly demonstrating the fact that electron–phonon coupling strength is diminishing in case of coating of Cr. The peaks at 525 and 564 cm^−1^ can be assigned to 2LA and A_1_(LO) modes respectively.^78^ Additionally, the peak at 470 cm^−1^ can be assigned to the surface optical (SO) mode of ZnO.^80^ SO phonons may be generated at the interface between different materials with different dielectric constants and propagate along the interface. Their frequencies are located between the frequencies of the transverse optical (TO) and LO modes, and are expected to show a very dispersive behaviour regarding the dielectric constant of the outer medium.^81^ The increase in the intensity of the SO mode after Cr coating suggests the passivation of surface states with metal coating.^81^

**Fig. 10:**
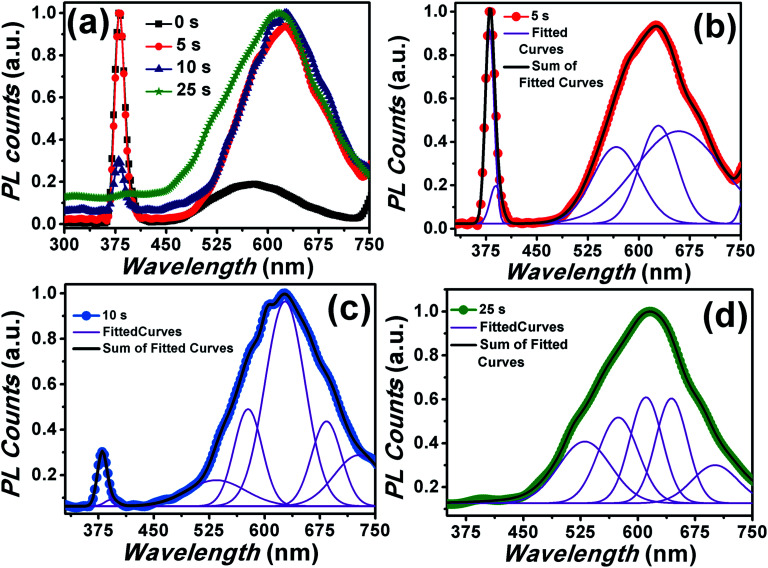
(a) PL spectra of Cr coated ZnO NRs annealed at 500 °C after Cr coating. (b–d) de-convoluted PL spectra of samples with Cr sputtering time 5, 10 and 25 seconds respectively.

Until now, we have focused on the effect of Cr coating over the passivation of DLE and simultaneous enhancement in the NBE towards UV only emission. Another interesting issue with ZnO nanostructures lies in their utilization for intense and broad visible region emission. The selective tuning of the visible emission in the ZnO is not well addressed in the literature. Here, we have performed the annealing of the Cr coated samples at 500 °C (shown in Fig. 10(a)) for selectively tuning the visible light emission. Interestingly, it can be seen that the UV emission intensity decreases upon annealing and becomes diminished for the samples with Cr coating for more than 25 seconds. In addition to this, broader peak encompassing visible region with increase in the intensity value was observed by increasing the Cr sputtering time. Thus, these results indicate that a supply of thermal energy after Cr-sputtering affects the UV emission intensity. The PL results, therefore, support our proposition that it is the temperature which plays a key role in the modulation of the emission properties. The occupation of oxygen vacancies with annealing by Cr ions and formation and oxygen interstitials can be assigned for the significant enhancement of the DLE.^82,83^Fig. 10(b–d) show the de-convoluted PL spectra of samples annealed at 500 °C after coating Cr for 5, 10 and 25 seconds respectively. Interestingly, an enhancement and broadening in the emission spectra corresponding to orange, red and NIR regions can be clearly seen. The peak at 630 and 685 nm can be assigned to oxygen interstitials and antisite defects.^7,75^ Conclusively, the post sputtering annealing has selectively enhanced the DLE with the increase in Cr sputtering time. The above mentioned enhancement of DLE in a controlled manner can be helpful for photo-catalytic activity and ZnO based LEDs emitting in the visible region of the spectrum.

### 3.3 Device characterization

**Fig. 11:**
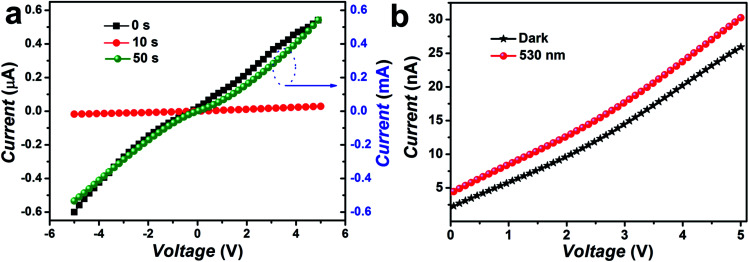
(a) The current (*I*)–voltage (*V*) characteristics of the pristine and Cr coated ZnO nanorods (b) Cr coated ZnO NRs based photodetector in the dark and under green light illumination.

The photo-response of pristine/Cr coated ZnO NRs has been studied under illumination using a lateral photodetector device configuration. *I*–*V* characteristics of ZnO and Cr coated ZnO devices were measured under dark and illumination of green light (*λ* = 530 nm and intensity = 5 mW cm^−2^) illumination are shown in Fig. 11. A typical characteristic of a photodetector was observed with an overall increment in the current level with the increasing bias in between the lateral Ag electrodes. In addition to this it was clearly observed that the dark current of the photoconductor was significantly reduced with Cr coating (for 10 seconds sputtering). This decrease in dark current can be attributed to the formation of Schottky contact at the interface of Cr and ZnO nanorods. The dark current values were observed to be 0.10, 0.02 μA for pristine and Cr (for 10 s sputtering) coated ZnO NRs respectively.^84^ In the dark, oxygen molecules generally get adsorbed on the surface of ZnO NRs and capture the free electrons [O_2_ + *e*^−^ → O_2_^−^] thus, a depletion layer with low conductivity is created near the surface.^85,86^ After covering with Cr particles, the surface states and the adsorption of oxygen molecules do not change due to that Cr particles do not cover the whole surface of the NRs and the contact region between Cr and ZnO is very small.^87^ Additionally, since the work function of ZnO (4.1 eV) is lower than that of Cr (4.5 eV), electrons will flow from ZnO to Cr which can lead to the formation of Schottky barrier at the interface. The negatively charged Cr nanoparticle depletes the carriers near the surface of ZnO nanowires. Notably, the width of depletion layer is related to barrier height, depletion region near the Cr nanoparticle is larger than that at defect and adsorbed oxygen site. The formation of the large depletion region is the main reason for the decrease in the dark current after covering with Cr nanoparticles. However, Cr coated samples with sputtering time of 50 s it was observed that the dark current level got increased and that can be assigned to the formation of continuity of Cr agglomerates. The results clearly showed that with Cr coating dark current level can be significantly reduced, thereby suggesting its potential for transistor applications also, as lower dark current level is required to get higher ON/OFF ratio.

Further, the photoconductivity of Cr coated ZnO NRs based device was found to be enhanced over the pristine ZnO device under the same measurement condition for visible light. Typically, ZnO is not sensitive for visible light therefore it lacks visible light detection. The origin of enhanced visible photo-response of Cr coated ZnO NRs was a result of the inter-band transition of Cr d-band electrons as an effect of the illumination of visible light.^84^ The filled d-band in Cr, below the Fermi level provides large number of electrons for interband transition under visible light illumination. Upon green light excitation, the d-band electrons of Cr atoms were excited to the conduction band with a final transfer to ZnO NRs due to the presence of electric field at the metal–semiconductor junction formed between Cr and ZnO. The combined effects of electrons transfer due to interband transition and plasmon resonance have resulted into increased photoconductivity in the green region.^84,88^

## 4. Conclusions

The NBE emission and DL emission of ZnO NRs are enhanced and suppressed, respectively, by Cr coating, which can be attributed to a combination of hot electron transfer from Cr-sp band to ZnO conduction band and recombination of electrons at defect levels with hot holes present at d band of Cr. Moreover, charge recombination can be assigned for suppression of the defect emissions corresponding to the 
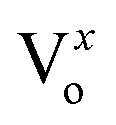
 and 
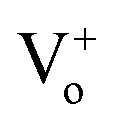
 assisted emissions in the case of Cr. Interband transitions present in the metal have been shown to have strong influence on the emission properties. KM absorption spectra have confirmed the interband transitions nearly at 1.56 eV for Cr. We further believe that the utilization of Cr plasmonics for passivation of defect states of ZnO NRs will play a significant role towards the development of highly efficient optoelectronic devices.

## Conflicts of interest

There are no conflicts to declare.

## Supplementary Material
